# Cognitive-motor network integration as a behavioral marker of cognitive reserve in post-stroke rehabilitation

**DOI:** 10.3389/fneur.2026.1802051

**Published:** 2026-06-09

**Authors:** Katharina Kreiger, Elisabeth Weiss, Felix Fluri

**Affiliations:** 1 Faculty of Psychology, University of Innsbruck, Innsbruck, Austria; 2 Kliniken Valens, Rheinburg Klinik, Walzenhausen, Switzerland; 3 Department of Neurology, University Hospital of Würzburg, Würzburg, Germany

**Keywords:** behavioral networks, cognitive reserve, cognitive-motor integration, functional recovery, post-stroke rehabilitation, stroke outcomes

## Abstract

**Background:**

Functional recovery after stroke varies substantially between individuals, even after standardized inpatient rehabilitation. Cognitive reserve is increasingly considered a key determinant of recovery potential, yet it is typically approximated using indirect proxy measures that may not capture the underlying functional mechanisms of recovery. Network-based approaches may provide a more mechanistic operationalization of cognitive reserve.

**Objective:**

This study investigated whether (a) traditional cognitive reserve proxies predict rehabilitation response, (b) responders differ from non-responders in respect to baseline cognitive performance structure, and (c) rehabilitation response is associated with greater cross-domain cognitive-motor network integration at admission.

**Methods:**

In this retrospective cohort study, 100 patients (≥ 65 years) with ischemic stroke were included. Functional outcomes were assessed using a battery of motor tests at admission and discharge. A responder was defined as someone who improved in at least two functional domains. Cognitive performance was assessed using the CERAD battery. Cognitive reserve proxies included years of education and engagement in leisure activity domains. Group differences and predictors of responder status were examined using regression models controlling for stroke severity (NIHSS).

**Results:**

Cognitive leisure activities emerged as the strongest predictor of responder status (OR = 4.84), whereas education and other leisure domains were not retained. Exploratory factor analysis revealed two baseline cognitive dimensions (Memory, Executive-Spatial), but responders did not show significantly higher baseline cognitive scores. Network analyses demonstrated a more integrated cognitive-motor architecture in responders, characterized by higher density and lower sparsity. Dexterity and delayed verbal recall showed the highest centrality in the responder network, alongside processing speed as a consistently contributing node across centrality indices.

**Conclusion:**

Post-stroke cognitive reserve may be less dependent on a high cognitive performance or demographic proxies but may be due to a more integrative organization of cognitive-motor functioning. Our findings support a network-based conceptualization of cognitive reserve with direct implications for integrative rehabilitation strategies.

## 1 Introduction

Stroke is a severe neurological condition, with up to 60% of survivors experiencing cognitive impairment (1) and up to 30% presenting at least moderate motor deficits (2). Despite standardized rehabilitation programs, there is substantial interindividual variability in functional recovery. Although established predictors such as initial stroke severity and lesion volume account for some of this variability, they do not fully explain the observed heterogeneity in outcomes. Increasing attention has therefore turned to the concept of cognitive reserve and to network-level brain mechanisms as potential determinants of post-stroke recovery.

Cognitive reserve is defined as the brain’s capacity to optimize or maximize performance through differential recruitment of brain networks or alternative cognitive strategies, allowing individuals to maintain cognitive function despite age-related or disease-related brain changes or pathology (3–7). Cognitive reserve is commonly estimated indirectly using proxy indicators such as years of education, occupational complexity, and engagement in cognitively stimulating leisure activities (4, 7, 8). Although these measures capture aspects of lifelong cognitive enrichment, they do not directly represent the functional mechanisms through which reserve may manifest. It is hypothesized that individuals with higher cognitive reserve may have greater potential for reorganization of neural processing following stroke, thereby achieving greater functional improvement. Previous studies suggest that cognitive reserve moderates the association between lesion characteristics, functional connectivity, and recovery potential (9). Importantly, cognitive reserve is not equivalent to baseline cognitive performance but rather reflects the efficiency of cognitive resources in compensating for deficits due to brain pathology (3, 10, 11). Proxy measures such as education or leisure activities provide only indirect approximations of this latent construct and may capture heterogeneous mechanisms (8, 12–14). In the present study, we therefore treat cross-domain behavioral network integration rather as a hypothesized correlate of reserve-related efficiency and less as a direct measure of cognitive reserve (15, 16). In parallel, recent advances in neuroscience research indicate that stroke should not only be conceptualized as focal tissue damage, but as a disorder of large-scale brain networks (17). Certain strategically important hub regions contribute disproportionately to global network efficiency and lesions involving such hubs are associated with poorer functional outcomes independent of lesion volume (18–20). Importantly, outcome appears to depend less on the isolated damage of single regions than on the integrity and reorganization of distributed network interactions (19, 20). Functional recovery has therefore been linked to the restoration of network connectivity (21) and, in particular, to the temporal organization of network activation dynamics (22). From this perspective, post-stroke improvement in cognitive and motor function results from adaptive reorganization within motor, sensory, and cognitive networks rather than from the size of stroke lesion alone (17, 23).

These converging lines of evidence suggest that cognitive reserve may itself be a network-level phenomenon characterized by efficiency, flexibility, and compensatory capacity. However, it remains unclear whether such network efficiency can be operationalized directly rather than inferred solely from proxy measures. Additionally, little is known, which specific cognitive functions may represent key integrative network nodes supporting recovery. Understanding these mechanisms is essential for developing targeted rehabilitation strategies that strengthen network-level functioning.

Hence, the present retrospective study examined whether (a) established proxy indicators of cognitive reserve (education and leisure activities) predict rehabilitation response, (b) responders differ from non-responders in the latent structure of baseline cognitive performance rather than simply showing higher baseline scores, (c) responders and non-responders differ in cross-domain cognitive-motor network integration at admission.

## 2 Methods

### 2.1 Study design and sample

This retrospective cohort study included patients with ischemic stroke who underwent inpatient neurorehabilitation between March 2024 and March 2025. All patients were treated with a standardized inpatient rehabilitation program consisting of a comparable scheduled therapy intensity (approximately 540 min per week). Rehabilitative measures comprised physiotherapy, occupational therapy, and neuropsychological therapy, supplemented by upper- and lower-limb group therapy. While therapy intensity was comparable across the cohort, the therapeutic focus and content were individualized based on functional impairments. Only study patients with complete data sets were included, defined as patients with both admission and discharge functional assessments. Additional inclusion criteria were age ≥ 65 years and a minimum inpatient stay of 1 week. In total, 100 patients met these criteria and were included in the analyses.

### 2.2 Measures

Stroke severity at admission was quantified using the National Institutes of Health Stroke Scale (NIHSS). Baseline cognitive performance was assessed using the CERAD neuropsychological battery. Functional mobility and endurance were evaluated at admission and discharge using the Timed Up and Go Test (TUG) and the 6-Minute Walk Test (6MWT). Upper-limb function was assessed using handgrip strength measured with a Jamar dynamometer and manual dexterity measured with the Box and Block Test (BBT). Upper-limb assessments were conducted bilaterally. All functional outcome measures were administered at both admission and discharge to capture rehabilitation-related change. All functional and cognitive test scores were converted to age-adjusted z-scores based on published normative data. CERAD scores were additionally corrected for educational level according to standard scoring procedures. Basic stroke characteristics (lesion laterality) were extracted from clinical records and used for descriptive purposes.

### 2.3 Definition of responders and non-responders

For all functional outcome measures, change scores were calculated as the difference between discharge and admission values. The primary aim of the study was to identify patients who demonstrated enhanced functional gains during inpatient rehabilitation while accounting for baseline performance differences. Baseline-adjusted improvement was quantified using residualized change scores. Specifically, for each outcome, a linear regression model was fitted in the full sample with baseline performance as predictor and the observed change score as dependent variable. The predicted change was derived from the model, and the residual was calculated as the difference between observed and predicted change, such that positive residual values indicated greater-than-expected improvement and negative values indicated poorer-than-expected improvement. Patients were classified as responders on the domain level if they demonstrated greater-than-expected improvement (positive residual values, residual > 0) and simultaneously exceeded published minimal clinically important difference (MCID) thresholds. MCID thresholds were defined as follows: for the 6MWT, 44 m for patients with baseline gait speed < 0.4 m/s and 71 m for patients with baseline gait speed ≥ 0.4 m/s (24); for the TUG, 2 s (25); for handgrip strength (Jamar), 5.5 kg (26); and for the BBT, 5.5 blocks (27). An overall responder status was assigned to patients who met the responder criteria in at least two functional outcome domains; all remaining patients were categorized as non-responders.

### 2.4 Cognitive reserve

Cognitive reserve was estimated exploratorily using proxy indicators including years of formal education and engagement in regular leisure activities. Occupational complexity was not included because the majority of participants were retired. Leisure activities were extracted from routine clinical documentation obtained at admission (patient interview). Activities were coded as present/absent (yes/no) and categorized into five domains: cognitive (e.g., reading, puzzles, handicrafts, playing music), physical (e.g., walking, cycling, swimming, fitness training), social (e.g., meeting friends, group activities, volunteering), artistic/creative (e.g., painting, singing), and outdoor/nature-related activities (e.g., hiking, camping, gardening). Because several activities can involve overlapping components (e.g., group sports, playing music), individual activities could be assigned to more than one domain and were modelled accordingly as multi-categorical indicators.

### 2.5 Statistical analyses

All statistical analyses were conducted in RStudio (version 2025.05.1 + 513; Posit Software, PBC). Descriptive statistics are reported as mean (SD) or median (IQR), as appropriate. Group differences between responders and non-responders were examined using independent-samples t-tests or Mann–Whitney *U* tests for continuous variables and *χ*^2^ tests for categorical variables. Only complete cases with both admission and discharge functional assessments were included.

To examine whether cognitive reserve proxies predicted responder status, an exploratory forward stepwise logistic regression was performed with responder status (responder vs. non-responder) as the dependent variable. NIHSS at admission was included as a covariate. Predictors comprised years of education, the number of leisure activity categories, and engagement in leisure activity domains (cognitive, physical, social, artistic/creative, and outdoor). Model fit was evaluated using likelihood ratio tests (Δ*χ*^2^) and pseudo-*R*^2^ indices (McFadden and Nagelkerke). Regression coefficients are reported as unstandardized estimates (b) with standard errors and Wald tests.

The latent structure of baseline cognitive performance was examined using exploratory factor analysis (EFA) with Promax rotation and the minimum residual method. The EFA included all CERAD subtests administered at admission, covering verbal learning across three trials, delayed verbal recall and recognition, relative verbal retention, processing speed, cognitive flexibility, delayed visual memory and relative visual retention, visuoconstruction, and visual reasoning.

Sampling adequacy was assessed using the Kaiser–Meyer–Olkin (KMO) measure and Bartlett’s test of sphericity. The number of factors was determined using parallel analysis and eigenvalue criteria. Factor scores were estimated using the regression method. Group differences in factor scores were tested using independent-samples *t*-tests, and effect sizes were reported as Hedges’ *g* with bootstrapped 95% confidence intervals (5,000 resamples). To examine whether the association between cognitive leisure activities and responder status was statistically explained by baseline cognitive performance, an indirect-effect model was estimated with cognitive leisure activities as predictor, responder status as outcome, baseline CERAD total score as mediator, and NIHSS at admission as covariate. Direct and indirect effects were estimated using nonparametric bootstrapping.

Cognitive-motor networks were estimated separately for responders and non-responders based on cognitive and motor z-scores at admission using Gaussian graphical models (partial correlation networks). Network structure was estimated using the EBICglasso approach. Node importance was assessed using normalized centrality measures. Network organization was summarized using global network metrics (density, sparsity, average shortest path length, clustering coefficient, modularity, and edge weight variance). Between-group differences in global network structure were evaluated using the NetworkComparisonTest, including tests of global strength and network structure invariance. In addition, node-deletion analyses were conducted to examine the contribution of individual nodes to overall network organization, with stability assessed via bootstrapping (500 iterations).

A two-tailed significance level of *α* = 0.05 was applied. Given the exploratory nature of the study, *p*-values were interpreted descriptively.

## 3 Results

### 3.1 Sample characteristics

Data from 100 in patients with ischemic stroke were analyzed. Mean age was 77.2 years (SD = 6.6; range 65–91 years), and the average inpatient stay was 30 days (SD = 13; range 9–68 days). Forty-nine participants were female and 51 male. Based on the predefined criteria, 56 participants were classified as responders and 44 as non-responders. Responders and non-responders did not differ significantly in age (*t*(98) = 0.67, *p* = 0.50), length of stay (*t*(98) = 1.07, *p* = 0.29), or sex distribution (*χ*^2^(1, *N* = 100) = 0.03, *p* = 0.86). Baseline stroke severity (NIHSS at admission) was comparable between groups (median [IQR]: 2.5 [1.75–5.0] vs. 2.5 [1.0–4.0]; Mann–Whitney U = 1,341, *p* = 0.45). Bilateral lesions were rare (*n* = 9) and excluded from laterality analyses; lesion laterality (left vs. right hemisphere) did not differ between responders and non-responders (*χ*^2^(1, *n* = 91) = 0.01, *p* = 0.92). Baseline functional performance was largely comparable between groups, with the exception of TUG at admission, where non-responders showed longer completion times than responders (*t*(99) = 2.13, *p* = 0.036); no significant differences were observed for 6MWT (*t*(99) = 0.41, *p* = 0.681), handgrip strength (*t*(99) = −0.64 *p* = 0.524), or BBT (*t*(99) = −0.86, *p* = 0.393). Baseline cognitive performance (CERAD total z-score) did not differ significantly between groups (*t*(98) = 1.85, *p* = 0.068), with both groups performing below age-adjusted norms. The baseline clinical and stroke characteristics are listed in Table 1.

**Table 1:** Demographic data and baseline clinical and stroke characteristics.

Characteristic	Non-responder (*n* = 44)	Responder (*n* = 56)
Age, years, mean (SD)	77.7 (7.2)	76.8 (6.2)
Range	65–89	65–91
Female sex, n (%)	22 (50.0)	27 (48.2)
Male sex, n (%)	22 (50.0)	29 (51.8)
Length of inpatient stay, days, mean (SD)	31.8 (11.7)	28.9 (14.4)
Range	13–56	9–68
NIHSS at admission, median (IQR)	2.5 (1.75–5.00)	2.5 (1.00–4.00)
NIHSS severity, *n* (%)		
Mild (0–4)	32 (72.7)	44 (78.6)
Moderate (5–15)	10 (22.7)	10 (17.9)
Severe (>15)	2 (4.5)	2 (3.6)
Lesion laterality, *n* (%)		
Left	21 (47.7)	29 (51.8)
Right	22 (50.0)	27 (48.2)
Functional scores at admission, mean (SD)		
6MWT (m)	266.7 (134.5)	254.7 (151.5)
TUG (s)	22.4 (16.5)	16.3 (12.6)
Handgrip strength (kg)	21.2 (6.5)	22.1 (7.0)
BBT (blocks)	64.6 (2.9)	65.0 (2.5)
Cognitive score at admission		
CERAD total (z-score), mean (SD)	−0.64 (0.80)	−0.99 (1.04)

NIHSS, National Institutes of Health Stroke Scale; 6MWT, Six-Minute Walk Test; TUG, Timed Up and Go Test; BBT, Box and Block Test; CERAD, Consortium to Establish a Registry for Alzheimer’s Disease neuropsychological battery. CERAD total scores are reported as age- and education-adjusted z-scores. Upper-limb assessments (handgrip strength, BBT) were conducted bilaterally, values reflect the mean of both sides at admission.

### 3.2 Cognitive reserve proxies predict rehabilitation response

**Figure 1:**
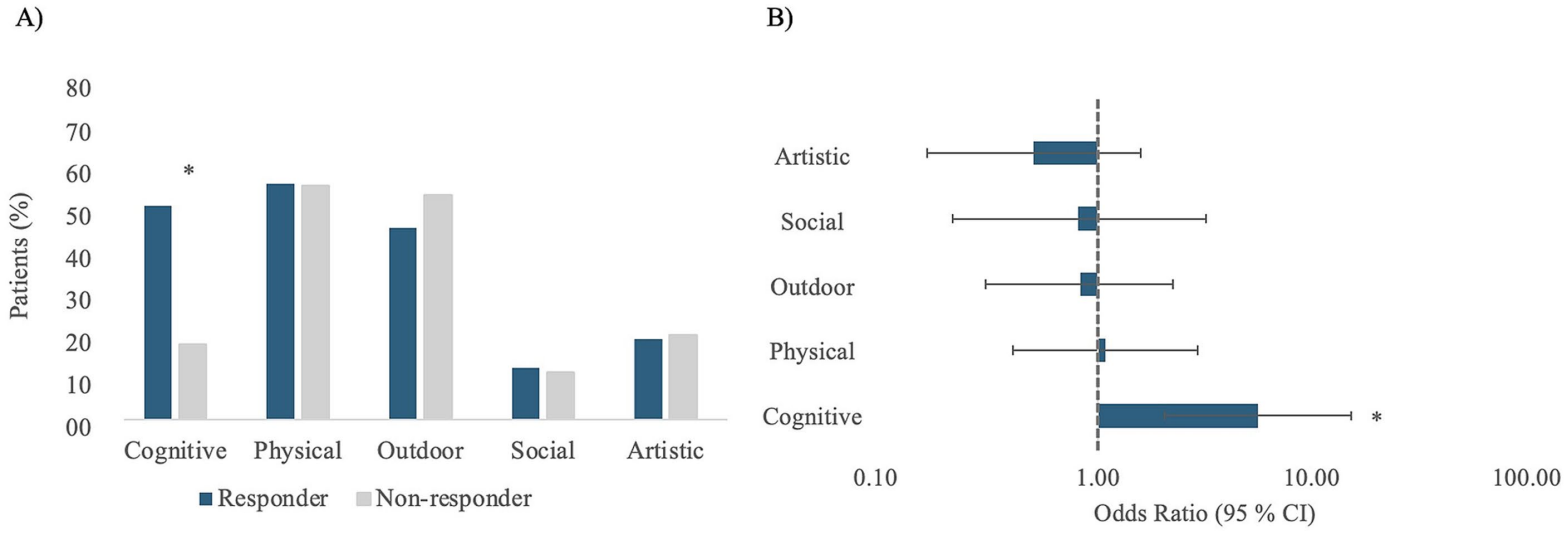
Leisure activity engagement and responder status. **Panel A** shows the proportion of patients engaging in each leisure activity domain by responder status. **Panel B** displays odds ratios (OR) with 95% confidence intervals from logistic regression prediction responder status, controlling for stroke severity (NIHSS) at admission. The dashed vertical line indicates OR = 1 (no effect). The *x*-axis in Panel B is on a logarithmic scale. *p* < 0.001.

On average, participants reported engagement in approximately two different activity categories, with comparable values in responders (*M* = 1.80) and non-responders (*M* = 1.77). Responders reported more frequently engagement in cognitive leisure activities (52%) compared with non-responders (18%), whereas the distribution of physical, social, artistic, and outdoor activities was broadly similar between groups (Figure 1, Panel A). The odds ratio for cognitive leisure activities as predictor of responder status is displayed in Figure 1, Panel B.

To test whether cognitive reserve proxies predicted responder status, a forward stepwise logistic regression was performed controlling for NIHSS at admission. Predictors included years of education, the number of leisure activity categories, and engagement in each activity domain (cognitive, physical, social, artistic/creative, and outdoor). The final model significantly improved fit compared with the null model (Δ*χ*^2^ = 12.51, *p* < 0.001), explaining 9.1% (McFadden *R*^2^) to 15.8% (Nagelkerke *R*^2^) of the variance in responder status. Cognitive leisure activities were retained as the only significant predictor (*b* = 1.58, SE = 0.47, Wald = 11.07, *p* < 0.001, OR = 4.84, 95% CI [1.91, 12.14]), whereas education and other leisure domains were not retained in the final model (Table 2).

**Table 2:** Logistic regression predicting responder status.

Predictor	*b*	SE	Wald	df	*p*
Intercept	−0.29	0.26	1.28	1	0.26
Cognitive activities	1.58	0.47	11.07	1	<0.001

Forward selection adjusted for NIHSS.

### 3.3 Baseline cognitive performance

To examine whether responders differed from non-responders in baseline cognitive functioning, an exploratory factor analysis (Promax rotation; minimum residual method) was conducted on CERAD measures at admission. Sampling adequacy was acceptable (KMO = 0.68) and Bartlett’s test of sphericity was significant (*χ*^2^(120) = 1509.47, *p* < 0.001). Parallel analysis supported a two-factor solution explaining 51.6% of the total variance. The first factor primarily reflected episodic memory and was labelled “Memory,” while the second factor reflected processing speed, cognitive flexibility, and visuospatial/visuoconstructive abilities and was labelled “Executive-Spatial” (see Supplementary Tables S1–S3).

**Figure 2:**
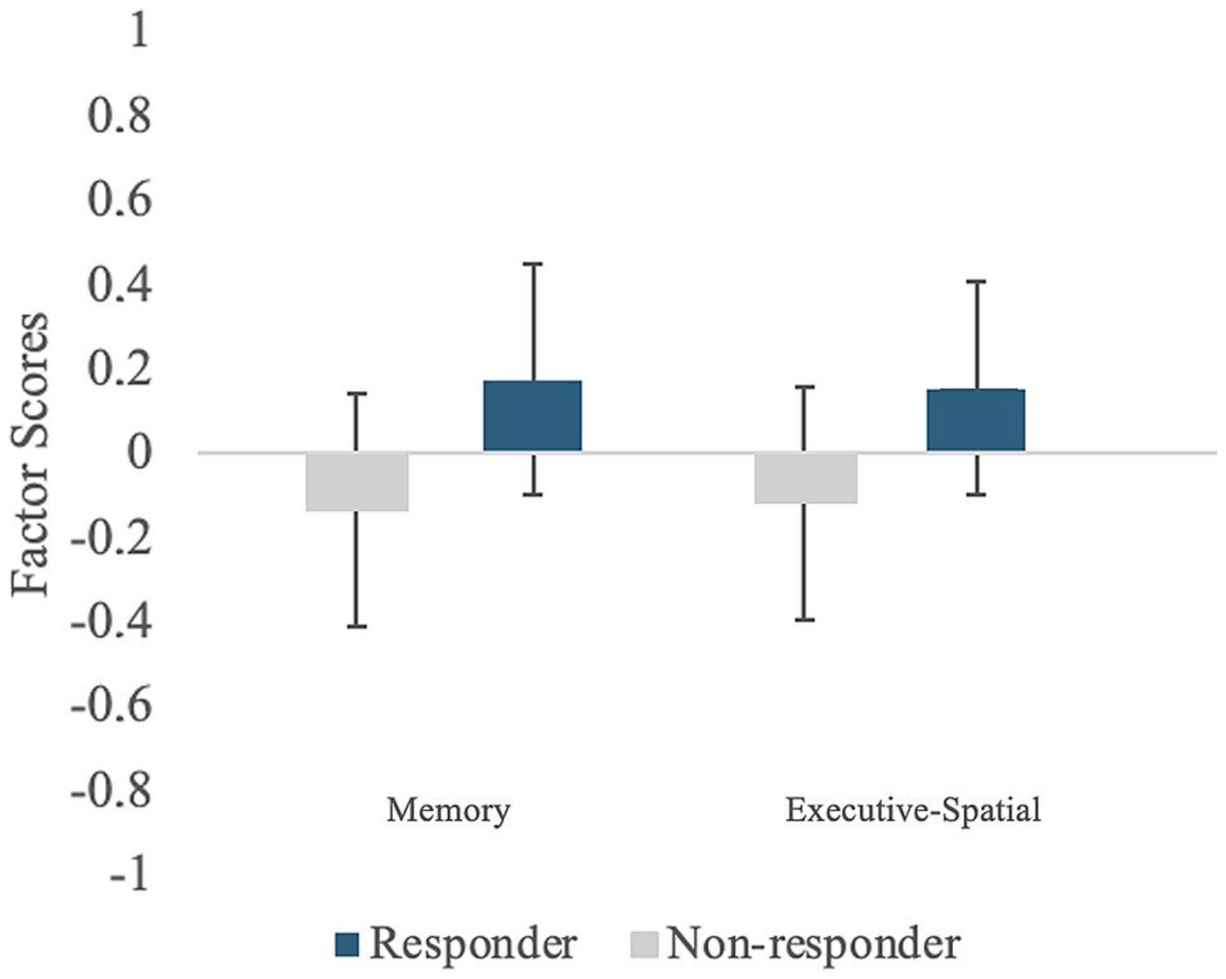
Baseline cognitive factor scores by responder status. Bars represent mean standardized factor scores with 95% confidence intervals for the memory factor (Factor 1) and the executive-spatial factor (Factor 2). Neither factor differed significantly between responders and non-responders (Memory: *t*(98) = 1.60, *p* = 0.11, *g* = 0.32; Executive-Spatial: *t*(98) = 1.76, *p* = 0.08, *g* = 0.34).

Responders and non-responders did not differ significantly in baseline factor scores (Memory: *t*(98) = 1.60, *p* = 0.11, *g* = 0.32, 95% CI [−0.06, 0.70]; Executive-Spatial: *t*(98) = 1.76, *p* = 0.08, *g* = 0.34, 95% CI [−0.03, 0.78], see Figure 2). To examine whether the association between cognitive leisure activities and responder status was explained by baseline cognitive performance, a mediation model was estimated with CERAD total score as mediator and NIHSS as covariate (Supplementary Figure S1). Cognitive leisure activities showed a significant direct association with rehabilitation response (*b* = 0.37, SE = 0.10, *z* = 3.91, p < 0.001), indicating that the relationship between cognitive leisure engagement and rehabilitation success was not dependent on higher baseline cognitive performance. The indirect effect via CERAD total score was non-significant (*b* = −0.02, SE = 0.02, *z* = −0.67, *p* = 0.50), suggesting that cognitive leisure activities predict responder status independently of baseline cognitive level.

### 3.4 Cognitive-motor network organization differentiates responders from non-responders

**Figure 3:**
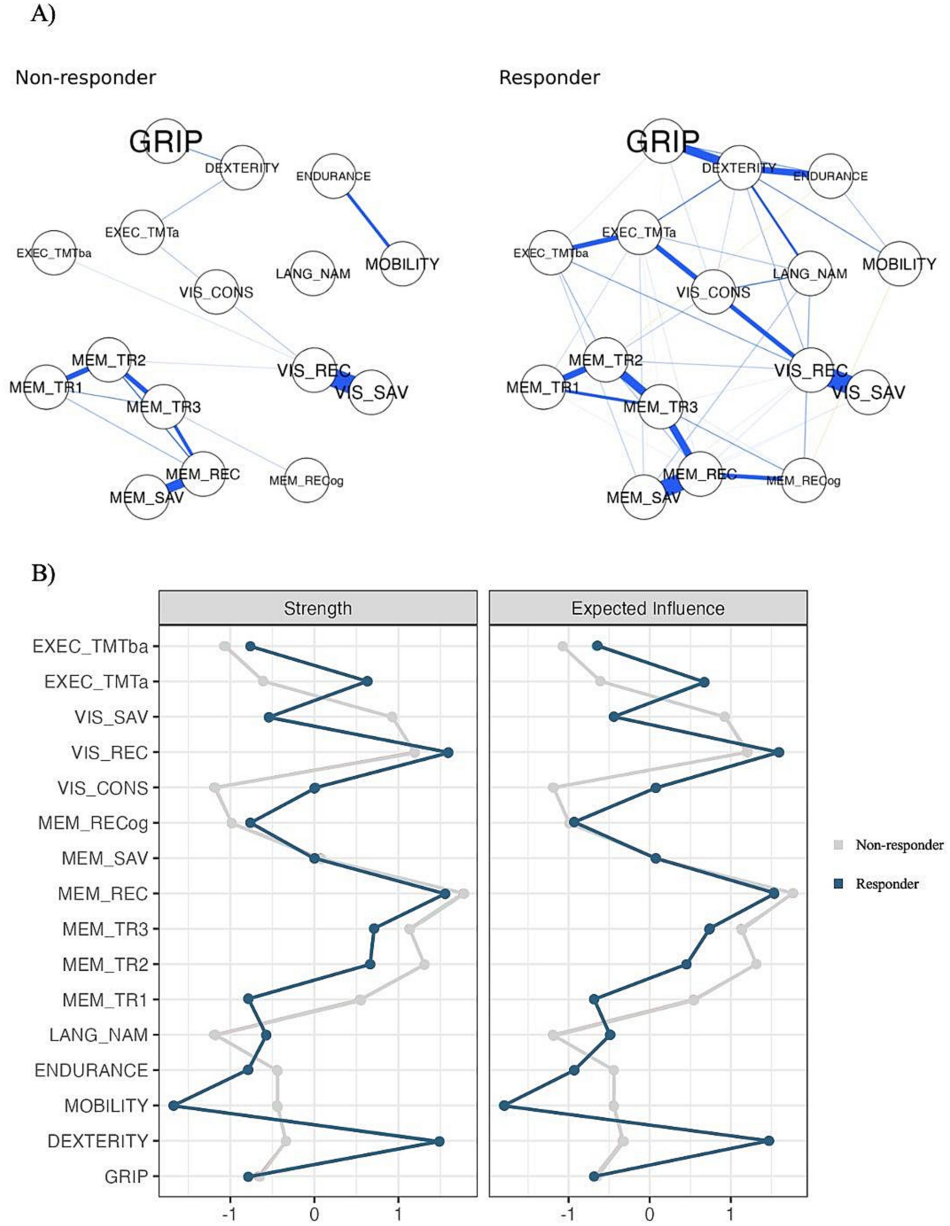
Cognitive-motor network organization and node centrality by responder status. **Panel A** shows network representations for non-responders (*n* = 44) and responders (*n* = 56). Nodes represent cognitive and motor variables; edges indicate partial correlations (blue = positive, red = negative correlations). Edge thickness reflects connection strength. **Panel B** shows normalized strength and expected influence centrality indices for each node separately for responders and non-responders. Motor nodes (GRIP, DEXTERITY, MOBILITY, ENDURANCE) are positioned at the bottom of Panel B. Positive centrality values indicate above-average node importance within the respective network. MEM_TR1–TR3 = verbal learning trials 1–3; MEM_REC = delayed verbal recall; MEM_RECog = verbal recognition; MEM_SAV = relative verbal retention; VIS_REC = delayed visual recall; VIS_SAV = relative visual retention; VIS_CONS = visuoconstructive abilities; EXEC_TMTA = Trail Making Test Part A (processing speed); EXEC_TMTB = Trail Making Test Part B (cognitive flexibility); LANG_NAM = object naming; GRIP = grip strength; DEXTERITY = manual dexterity; ENDURANCE = endurance; MOBILITY = mobility.

To test whether rehabilitation response was associated with more integrated cross-domain organization, network analyses were conducted separately for responders and non-responders. Global network indices indicated a more integrated network architecture among responders, reflected in significantly higher density and lower sparsity (Table 3). The remaining global metrics (mean path length, clustering coefficient, modularity, and edge weight variance) did not differ significantly between groups. Network visualizations are displayed in Figure 3, Panel A.

**Table 3:** Comparison of global network characteristics between responder and non-responder.

Metric	Responder *M* (95% CI)	Non-responder *M* (95% CI)	Δ*M*	95% CI	*p*
Density	0.68 [0.61, 0.75]	0.57 [0.49, 0.64]	0.11	[0.00, 0.21]	0.05
Sparsity	0.31 [0.25, 0.39]	0.43 [0.36, 0.51]	0.12	[0.01, 0.21]	0.05
Avg. shortest path	3.99 [3.59, 4.45]	4.51 [3.77, 5.45]	0.52	[−0.27, 1.46]	0.29
Clustering coefficient	0.10 [0.08, 0.13]	0.08 [0.06, 0.12]	0.01	[−0.05, 0.02]	0.38
Modularity *Q*	0.21 [0.13, 0.29]	0.21 [0.11, 0.31]	0.01	[−0.11, 0.13]	0.92
Edge weight variance	0.02 [0.01, 0.02]	0.02 [0.01, 0.03]	0.00	[−0.01, 0.01]	0.59

Bootstrapping with 500 iterations.

Node-level centrality analyses (Figure 3, Panel B) revealed differential patterns of node importance across groups. In the responder network, motor-cognitive bridging was more prominent with dexterity showing the highest strength and expected influence centrality, followed by delayed verbal recall (MEM_REC) and processing speed (TMT_A). Node-deletion analyses further indicated that removal of processing speed and visual delayed recall (VIS_REC) most substantially increased network sparsity in responders. In responders, upper-limb motor performance showed stronger associations with cognitive nodes compared to non-responders. Dexterity was associated with processing speed (*r* = 0.50, *p* < 0.001) and visual abilities (*r* = 0.35, p < 0.001), while handgrip strength showed associations with processing speed (*r* = 0.28, *p* = 0.004), cognitive flexibility (*r* = 0.24, *p* = 0.02), and visuospatial memory (*r* = 0.25, *p* = 0.01). These motor-cognitive associations were not observed to the same degree in non-responders.

## 4 Discussion

The present study yielded the following main findings. First, engagement in cognitively demanding leisure activities was the most robust behavioral indicator of rehabilitation success, remaining predictive even after controlling for baseline stroke severity. In contrast, years of education, the overall variety of leisure activities, and non-cognitive activity domains did not contribute to response prediction. Second, rehabilitation response was not explained by superior baseline cognitive functioning: although baseline CERAD performance could be summarized by two latent dimensions reflecting episodic memory and executive-spatial abilities, responders and non-responders did not differ significantly on these factors. Third, and most importantly, responders were characterized by a denser cognitive-motor network architecture with greater overall number of connections. Within this organization, processing speed emerged as a key integrative hub linking memory-, executive-spatial-, and motor-related nodes, with visual delayed recall also contributing to network connectivity.

### 4.1 Network-based mechanisms of cognitive reserve after stroke

The present findings suggest that cognitive reserve in stroke survivors may be more adequately captured through dynamic network properties than through static proxies alone, with cognitively stimulating leisure activities emerging as stronger predictors of rehabilitation success than educational attainment. This pattern suggests that active, lifelong cognitive engagement may be more critical for post-stroke recovery than early acquired, static factors such as formal education (28, 29). These results align with the “use-it-or-lose-it” hypothesis of cognitive reserve maintenance, which posits that ongoing cognitive stimulation throughout the lifespan builds adaptive capacity that can be mobilized following brain injury (11, 30). Contemporary models increasingly conceptualize cognitive reserve as a dynamic, evolving process rather than a fixed attribute (10, 31). Bettcher et al. (31) demonstrated that changes in cognitive reserve over time predict clinical outcomes more effectively than single time-point measurements, with maintenance of reserve attenuating the negative effects of brain atrophy. This framework is particularly relevant to stroke recovery, within which the capacity for network reorganization may determine functional outcomes rather than baseline structural integrity (32, 33). Our findings are consistent with this process-oriented view, suggesting that cognitive reserve may manifest in more efficient resource utilization, as indicated by the observed network density differences. Processing speed emerges as a critical hub linking cognitive and motor functions in stroke rehabilitation (34–36). Su et al. (36) demonstrated that slowed processing speed represents a central mechanism underlying post-stroke cognitive impairment across multiple domains, even after accounting for other cognitive factors. Our findings extend this work by indicating that processing speed is not merely a single cognitive function but a higher-level cognitive entity which reflects the efficiency with which information is transmitted across distributed brain systems. Higher levels of network integration appear to support faster and more coordinated information processing, which in turn facilitates functional recovery. In line with this view, Vecchio et al. (37, 38) reported that more efficiently organized brain networks in acute stroke patients were associated with better clinical outcomes, and that changes in network organization predicted subsequent rehabilitation success.

This suggests that cognitive reserve may be associated with network efficiency mechanisms, as indicated by the higher overall network density observed in responders, though the present behavioral data do not allow direct conclusions about information transfer between systems. Our findings collectively support the view that cognitive reserve after stroke is not primarily reflected in structural markers or static performance levels, but rather in the capacity of cognitive-motor networks to reorganize efficiently in response to injury (32, 34, 39). Andrushko et al. (34) demonstrated that improvements in processing speed following aerobic exercise were associated with decreased functional connectivity between the dorsolateral prefrontal cortex and sensorimotor networks. Although our study is limited to behavioral network data and does not permit direct conclusions about underlying neural mechanisms, the observed patterns are broadly consistent with the view that individuals with higher cognitive reserve may possess more adaptable functional architectures. Whether such behavioral network differences reflect genuine neural reorganization remains to be confirmed in future multimodal studies (32, 33, 39).

### 4.2 Implications

From a clinical perspective, these findings highlight the potential value of interventions that enhance cross-domain integration, rather than addressing cognitive or motor deficits in isolation. Rehabilitation programs incorporating cognitively demanding activities, processing speed training, and coordinated motor-cognitive practice may help strengthen network-level organization.

### 4.3 Limitations

Several limitations must be acknowledged. The retrospective design prevents causal inference, and the sample size limits the detection of small effects. In our sample, the majority of patients presented with mild stroke severity, which limits the generalizability of the findings to patients with moderate-to-severe stroke. Occupational complexity could not be included due to retirement status of most patients. Additionally, neuroimaging-derived connectivity metrics were not available; thus, the behavioral networks demonstrated here should be interpreted as functional correlates rather than direct measures of neural architecture. Future multimodal studies combining cognitive, behavioral, and connectivity data will be essential to validate these interpretations. A further limitation concerns the operationalization of cognitive leisure activities. Although coded as a cognitive domain, several activities within this category (e.g., handicrafts, playing music) involve substantial motor engagement and may therefore partly reflect motor reserve rather than purely cognitive reserve. The observed association between cognitive leisure activities and responder status may thus capture a broader form of cognitive-motor reserve rather than cognitive reserve in isolation. Future studies should consider more fine-grained categorization of leisure activities to disentangle cognitive and motor components.

## 5 Conclusion

Cognitive reserve after stroke appears to be reflected less in static demographic factors or isolated cognitive abilities and more in the dynamic efficiency and integration of large-scale networks. In particular, patients classified as responders (i.e., individuals showing greater-than-expected functional improvement during rehabilitation) demonstrated more densely interconnected cognitive-motor networks, with dexterity and delayed verbal recall emerging as nodes with particularly high centrality. These findings converge with neuroimaging evidence implicating hub regions such as the default mode network and thalamus in recovery processes (40, 41) and are consistent with a conceptualization of cognitive reserve as an emergent, network-based capacity that may facilitate adaptive reorganization following brain injury. Furthermore, regular engagement in cognitively demanding activities emerged as a behavioral marker associated with rehabilitation success, underscoring the potential importance of lifelong cognitive engagement for resilience and recovery after stroke.

## Data Availability

The raw data supporting the conclusions of this article will be made available by the authors, without undue reservation.
