# Supplementary material for: Cognitive-motor network integration as a behavioral marker of cognitive reserve in post-stroke rehabilitation
Source: Front Neurol. 2026 Jun 9;17:1802051. doi: 10.3389/fneur.2026.1802051 (PMC13286753; doi:10.3389/fneur.2026.1802051)
Supplement: Supplementary file 1 [file Table_1.DOCX]

Supplementary Material

# Supplementary Figures and Tables

**Supplementary Table S1.** Assessment of Factorability Assumptions

| Test | Value | df | p |
| --- | --- | --- | --- |
| Kaiser-Meyer-Olkin (KMO) | 0.680 | - | - |
| Bartlett’s Test auf Sphericity | 1509.47 | 120 | < .001 |

**Supplementary Table S2.** Factor Characteristics

| Factor | Eigenvalue | Variance Explained (%) | Cumulative (%) |
| --- | --- | --- | --- |
| 1 | 6.99 | 28.3 | 28.3 |
| 2 | 2.07 | 23.3 | 51.6 |

**Supplementary Table S3**. Factor loadings after Promax-Rotation

| Function | Description | Factor 1 | Factor 2 | Uniq. |
| --- | --- | --- | --- | --- |
| Verbal learning performance  _MEM_TOTAL_ | *Total number of learned words across three trials* | 0.94 |  | 0.11 |
| Verbal learning capacity  _MEM_TR3_ | *Recallable words in the third learning trial* | 0.89 |  | 0.24 |
| Verbal long-term memory  _MEM_REC_ | *Delayed free recall* | 0.88 |  | 0.25 |
| Verbal learning ability  _MEM_TR2_ | *Recallable words in the second learning trial* | 0.78 |  | 0.28 |
| Verbal supraspan  _MEM_TR1_ | *Recallable words after one learning trial* | 0.72 |  | 0.47 |
| Verbal recognition  _MEM_RECog_ | *Recognition memory with cues* | 0.64 |  | 0.69 |
| Relative verbal retention  _MEM_SAV_ | *Ratio of recall to learning performance* | 0.49 |  | 0.69 |
| Cognitive flexibility  _EXEC_TMTb_ | *Speed of attentional switching* |  | 0.95 | 0.19 |
| Processing speed  _EXEC_TMTa_ | *Basic information processing speed* |  | 0.82 | 0.43 |
| Executive control  _EXEC_TMTba_ | *Relative index between TMT A and B* |  | 0.79 | 0.47 |
| Visual memory  _VIS_REC_ | *Delayed visual recall* |  | 0.77 | 0.37 |
| Relative visual retention  _VIS_SAV_ | *Ratio of visual recall to learning phase* |  | 0.59 | 0.64 |
| Visuoconstruction  _VIS_CONS_ | *Copying figures* |  | 0.49 | 0.69 |
| Visual reasoning  _LANG_NAM_ | *Inferring and naming figures* |  | 0.42 | 0.77 |

Note. All cognitive measures were derived from the CERAD neuropsychological test battery. Factor loadings ≥ 0.40 are reported. Verbal fluency and intrusion errors were excluded due to ambiguous assignment to a single cognitive domain. MEM = verbal memory subtests (word list learning, recall, and recognition); EXEC = executive function measures derived from the Trail Making Test (TMT); VIS = visuospatial and visuoconstructive subtests; LANG = language-related subtests. TMT A = Trail Making Test Part A; TMT B = Trail Making

Test Part B; Uniq. = uniqueness.

## Supplementary Figures


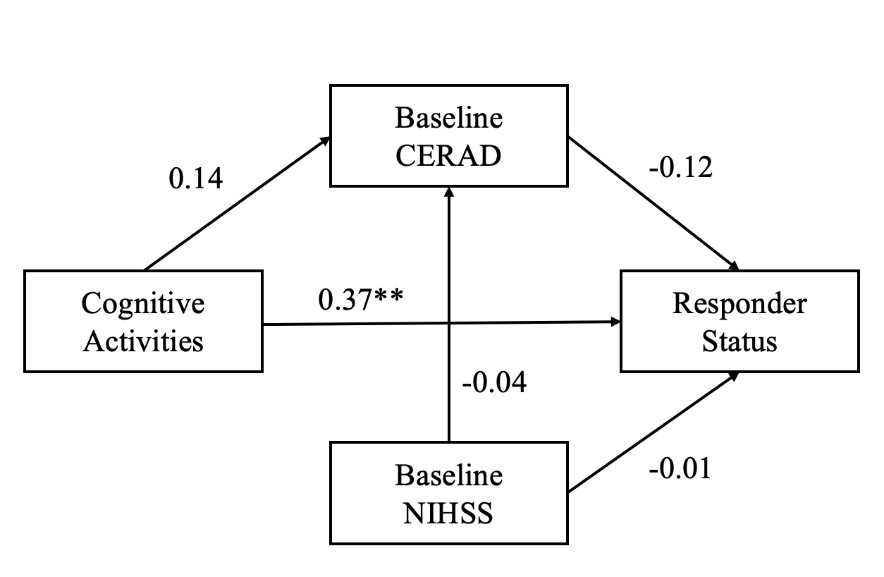


**Supplementary Figure S1.** Mediation model examining the association between cognitive leisure activities and global rehabilitation response. Baseline CERAD total score was specified as a mediator and baseline NIHSS as a covariate. Path coefficients represent standardized estimates.

p < .05*, p < .01**, p < .001***.

.
